# Non-Invasive Sensing of Nitrogen in Plant Using Digital Images and Machine Learning for *Brassica Campestris* ssp. *Chinensis* L.

**DOI:** 10.3390/s19112448

**Published:** 2019-05-29

**Authors:** Xin Xiong, Jingjin Zhang, Doudou Guo, Liying Chang, Danfeng Huang

**Affiliations:** School of Agriculture and Biology, Shanghai Jiao Tong University, Shanghai, Shanghai 200240, China; xiongxin1989@sjtu.edu.cn (X.X.); jj.zhang@sjtu.edu.cn (J.Z.); adoutx@sjtu.edu.cn (D.G.)

**Keywords:** visible light imaging, phenotyping, machine learning, nitrogen nutrition index, leafy vegetable, precision fertilization

## Abstract

Monitoring plant nitrogen (N) in a timely way and accurately is critical for precision fertilization. The imaging technology based on visible light is relatively inexpensive and ubiquitous, and open-source analysis tools have proliferated. In this study, texture- and geometry-related phenotyping combined with color properties were investigated for their potential use in evaluating N in pakchoi (*Brassica campestris* ssp. *chinensis* L.). Potted pakchoi treated with four levels of N were cultivated in a greenhouse. Their top-view images were acquired using a camera at six growth stages. The corresponding plant N concentration was determined destructively. The quantitative relationships between the nitrogen nutrition index (NNI) and the image-based phenotyping features were established using the following algorithms: random forest (RF), support vector regression (SVR), and neural network (NN). The results showed the full model based on the color, texture, and geometry-related features outperforms the model based on only the color-related feature in predicting the NNI. The RF full model exhibited the most robust performance in both the seedling and harvest stages, reaching prediction accuracies of 0.823 and 0.943, respectively. The high prediction accuracy of the model allows for a low-cost, non-destructive monitoring of N in the field of precision crop management.

## 1. Introduction

Nitrogen (N) is one of the critical factors limiting crop nutrient and productivity and is a main environmental pollution factor in farmland [1,2]. An excessive amount of N fertilizer is often applied to ensure adequate yield and profitability from vegetable cultivation [3]; however, most of the N accumulates in the soils or is lost by runoff or lixiviation [1,4]. An accurate nutrition diagnosis and a timely and appropriate application of N fertilizer have been the focus in modern agriculture. 

Greenwood et al. [5] defined the critical N concentration (Nc) as the minimal concentration of total N in shoots that produce the maximum aerial dry matter (DM). A unique critical N dilution curve (CNC) was obtained by plotting these concentrations against the accumulated shoot biomass. Many studies have been conducted on the CNC of crops such as rice, wheat, barley, and maize [6,7,8,9]. The nitrogen nutrition index (NNI), i.e., the ratio of the actual N concentration of shoot dry matter to the critical N concentration, has been an effective and established indicator of crop N status, with deviations from the optimal values of NNI = 1 indicating N deficiency (NNI < 1) or N surplus (NNI > 1) [10]. The NNI not only directly reflects the surplus/deficit state of N nutrition, but can also be used to assess N requirement. However, conventionally, the NNI has been determined based on a chemical analysis method. This method is time-consuming and labor-intensive, as the measurement requires a specific analyst and laboratory equipment to determine the actual N concentration. 

Nutrition diagnosis is an important part of the scientific fertilization of crops. Recently, significant progress has been made in evaluating N nutrition status through alternative non-destructive methods. For example, the leaf chlorophyll content has been correlated to leaf N content [11]; usually, chlorophyll meters, such as the soil plant analysis development (SPAD), have been used to diagnose N status [12,13,14]. However, this has not been extensively applied to N management of crops, largely because of the lack of sensitivity and specificity, the intense sampling effort required, and the lack of generalized relationship with actual N fertilizer requirements. The spectral remote-sensing analysis technology has been significantly developed for the large-scale monitoring of plant nutrition [15,16]. However, it is difficult to capture and quantify micro-symptoms using a hyper-spectrometer [17]. Additionally, the data processing and computation processes are challenging [18], restricting its application. Chlorophyll fluorescence technology based on the detection of both chlorophyll and flavonols has been also reported to evaluate the plant N nutrition status recently [19,20,21]. For example, Padilla et al. [22] reported strong relationships between three fluorescence indices (leaf chlorophyll, flavonols, and N balance index) and the N content in cucumber; Similarly, Agati et al. [23] found leaf N percent was strongly correlated to flavonols and N balance index in white head cabbage. However, the N nutrition detection using this technology is limited because of the high cost of fluorescence-based sensors. 

The imaging technology based on visible light which is rapid, non-destructive, repetitive and relatively inexpensive, compared with optical techniques mentioned above, provides an approach to non-invasive sensing of nitrogen in plant. With the development of digital cameras and image-processing technology, they have been used in many fields such as in plant nutrition research. For example, various studies have reported the discrimination of plant mineral nutrient status based on imaging technology and plant phenotypic characteristics [24,25,26]. Many spectral indices in the visible region have been proposed for segmenting crop canopy images, specifically oriented towards green segmentation. Visible spectral-index based indices, including excess green index (ExG), excess red index (ExR), color index of vegetation extraction (CIVE), excess green minus excess red index (ExGR), and vegetative index (VEG) [27,28,29,30,31]. ExG, ExGR, CIVE and VEG also have been applied under a combined form in Guijarro et al. (2011) gaining in performance with respect to their individual application [32]. All these approaches need to fix a threshold for final segmentation. The ExG index (2G-R-B), which increases G value and decreases R and B values, achieved the best accuracy in the vegetation extraction [33,34]. The ExG has been most commonly used to segmentation the vegetation from the background. 

The goal of plant imaging and analysis is to measure the physiological, growth, development, and other phenotypic properties of plants through automated processes. Many of the technologies developed for image processing and analysis can be applied to plant phenotyping [18,35,36]. The phenotypic traits of plants, such as the color and morphology of the leaf, help indicate the plant nutrient and health status, which is closely related to the plant N content. Under N deficiency, plants appear to be weak and lack vigor and have fewer blades and smaller leaf area and lower leaf perimeter. Moreover, the leaf color becomes light green and chlorotic. Under N surplus, the leaves become dark green and exhibit hypertrophic and elongated internodes. The phenotyping based on imaging technology has been employed as an alternative method to evaluate plant N nutrition, but most research was only focused on color traits [37,38]. 

Machine learning (ML) has a good performance in efficiently discovering patterns and governing discovery from large datasets by simultaneously considering a combination of factors [39]. This is particularly useful in the case of plant growth development, where it is challenging to efficiently model the holistic effect of genetic, physiological, phenotypic, agronomic, meteorological, and human factor features on the plant. ML technology has been applied to the identification [40,41], classification [42,43], quantification [44,45], and prediction [46,47] of stress phenotyping in plants. In summary, ML approaches are typically useful in situations where large amounts of data are available, relating inputs (e.g., phenotypic data) to output quantities of interest (e.g., NNI).

Pakchoi (*Brassica campestris* ssp. *chinensis* L.) is one of the most popular vegetables, cultivated over a wide area from the northern to southern regions of China. The residual soil N in pakchoi production has been reported to be high enough to avoid the requirement of additional N in the subsequent season [4]. In the present study, an industrial camera was used in the visible light range to track the growth and N status of pakchoi under different N application rates. The images of pakchoi were captured from the top view, and the information in the two-dimensional images was quantified. The phenotypic features (color, texture, and morphology-related traits) were then extracted. An NNI quantitative evaluation model based on phenotyping was developed using three machine learning algorithms: random forest (RF), support vector regression (SVR) and neural network (NN). This study is expected to contribute toward a non-destructive, easy, low-cost diagnosis of plant N.

## 2. Materials and Methods

### 2.1. Experimental Design

The experiment was carried out in a glass greenhouse at the School of Agriculture and Biology, Shanghai Jiao Tong University, China. The pakchoi (cv. Hua wang) were cultivated in a substrate containing available N (332 mg·k^−1^), phosphorus (1.24 mg·kg^−1^), potassium (118 mg·kg^−1^), and organic matter (270.3 g·k^−1^). The seeds were sowed in a plug tray on 18 November 2017. After a nursing of 18 days, the seedlings were transplanted to pots (volume of 1.47 L) with one plant per pot. The N treatments (CK, T1, T2, and T3 corresponding to N levels of 0, 0.134, 0.163, and 0.191 g N·pot^−1^, respectively) were applied to each pot after one week of transplanting. The T3 treatment was based on the conventional N application rate. Three blocks were considered with 20 repetitions per block for 240 plants. Urea (Sinopharm Chemical Reagent Co., Ltd, Shanghai, China) was chosen as the N fertilizer. The urea was divided into six parts and applied every seven days. KH_2_PO_4_ (Sinopharm Chemical Reagent Co., Ltd, Shanghai, China) was selected as the phosphate and potassium fertilizer. The dosages of P and K in each treatment were the same, and the additive amount was 0.4613 g of KH_2_PO_4_ per pot. The substrate water content was controlled at a maximum water holding capacity of 75 ± 5% using the weighing method. The images and plant tissue were collected at different growth stages: G0, G1, G2, G3, G4, G5, and G6 corresponding to 0, 7, 14, 21, 28, 35, and 42 days after the first N application, respectively. 

The greenhouse environmental data were monitored every five minutes using an automatic data logging system (PM-11 Phytomonitor, Bio Instruments S.R.L., Chisinau, Moldova). During the experimental period, the air temperature ranged from −0.70 to 37.95 °C with an average of 13.88 °C. The average relative humidity was 86.4%, reaching a maximum of 99.2% and a minimum of 29.5%. The mean solar radiation in the greenhouse was 1.69 MJ·m^−2^·day^−1^.

### 2.2. Image Acquisition and Analysis

**Figure 1:**
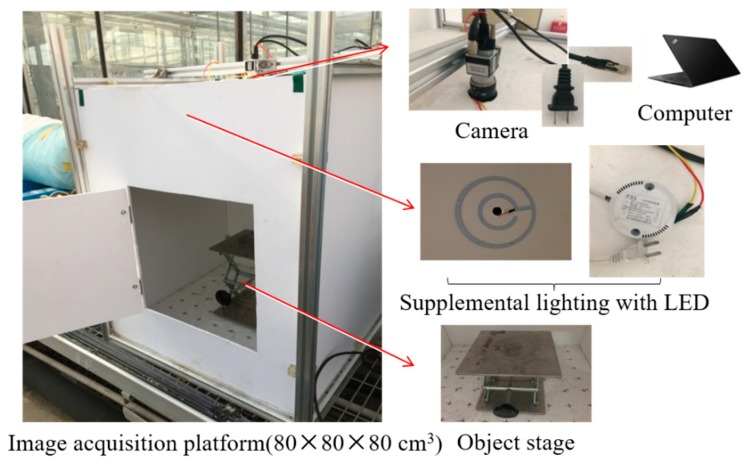
Image acquisition platform.

A self-made platform, i.e., a sealed box (80 × 80 × 80 cm^3^), with a set of camera and supplement light was used for image acquisition (Figure 1). An industrial camera (MV-EM1400C, MicroVision, Shaanxi, China) was mounted at the top center of the box, and a lens with a 12 mm focal length (M1214-MP2, MicroVision, Shaanxi, China) was implemented. Ring-shaped light emitting diodes (LEDs) (illumination: 6341 lux, the correlated color temperature (CCT): 6138K) as the sole light source were mounted on top of the box and centered on the camera. The pakchoi were sent to the platform one by one, and their images were taken from the top view. The aperture and exposure time of the camera were set to F16 and 0.13 s, respectively, throughout the experiment. We collected 29 plant images before N treatment. After N application, the images were acquired from G1 to G6 growth periods with 13–16 replicates for each nitrogen level in a given period. As a result, the total of the plant images collected was 382. The images were stored in an uncompressed PNG format to avoid color artifacts due to compression algorithms.

**Figure 2:**
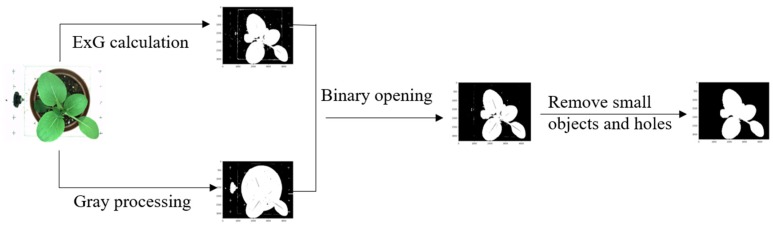
Image processing flow.

**Figure 3:**
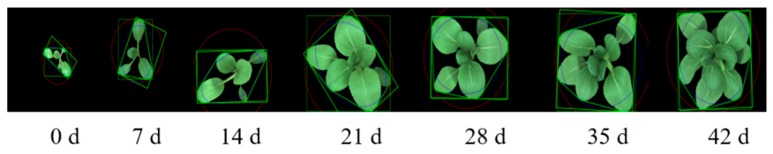
The pakchoi images at different growth periods (0, 7, 14, 21, 28, 35, and 42 days after the first N application, respectively).

The images analysis was performed using the OpenCV (3.4.4 version) and Python language (3.6.1). The details are given in the supplementary material section. The image processing included the computation of excess green index (ExG) and grayscale [48,49]. Figure 2 shows the process flow, and the details include: (1) ExG calculation: the original images were transformed by using 2G-R-B, and the two thresholds (where 40 identified the minimum value and 200 the maximum value) were set to identify the green part of the images (including plant and the green area of background). (2) Grayscale processing: the original images were pretreated by graying, and then the threshold value segmentation was used for removing the high brightness area. The grayscale value was set as 240. (3) Intersection calculation: we take the intersection of the mask obtained by the two operations above, and then the noise (small size) was eliminated based on the mathematical morphological open operation. (4) Denoising: the operations of denoising were done by using OpenCV. Removing objects smaller than the one tenth of the mask area was to remove the residual; filling contiguous holes smaller than one-twentieth of the mask area was to restore leaf veins deleted. Hence, the final images were acquired, and they are shown in Figure 3.

An extensive list of phenotypic features, including color, texture, and geometry, were extracted. The extraction of the color features involved the transformation of RGB (Red, Green, Blue), LAB (L is luminosity, A is the range from magenta to green, and B is the range from yellow to blue, and HSV ( Hue, Saturation, Value) color spaces. The texture attributes were extracted based on the grey co-occurrence matrix algorithm. The equations used were obtained from the study by Haralick and Shanmugam [50] and are described in detail in the supplementary material section. The geometrical features, such as the convex hull and boundary box, were extracted based on the plant contour. Table 1 lists the descriptions of the phenotypic features. 

### 2.3. Yield and Quality Measurement

The fresh weight and quality, i.e., chlorophyll, flavonoid, nitrate, and soluble protein, were measured at the last harvesting, with data representing the mean of three replicates (three plants per replication) per treatment. 

No. 2 leaves counted from the tip of the shoots were chosen to measure the quality traits. Dualex SCIENTIFIC+TM (FORCE-A, Orsay, France) was used for the chlorophyll and epidermal flavonoid measurements. The chlorophyll was used as given by the device because it was calibrated in μg·cm^−2^ units. The absorbance in the ultraviolet (UV) region at 375 nm due to the presence of flavonol in the epidermis was used as given by the device without transformation. The soluble protein content was assayed using the Coomassie brilliant blue method, whereas the nitrate content was measured based on the method given by Xiong et al. [51]. 

### 2.4. Nitrogen Concentration Determination 

The fresh shoot samples were heated at 105 °C for 20 min and dried at 75 °C for 72 h in an air-dry oven (DHG-9030A, Hangzhou, China). The dried samples were ground and sieved (60 mesh), and the nitrogen concentration was measured using Elementar (ELIII, Hanau, Germany). 

### 2.5. Nitrogen Nutrition Index (NNI) Calculation

The data were analyzed to determine Nc based on the method proposed by Justes et al. [52]. First, the analysis of variance (ANOVA, SPSS-16 software package) was used to identify the data points for which N does not limit growth (non-N-limiting) or when it is not in excess (N-limiting) from the experimental data. The N-limiting growth treatment is defined as a treatment in which a supplement of N application leads to a significant increase in the shoot biomass. The non-N-limiting growth treatment is defined as a treatment in which a supplement of N application does not lead to an increase in the shoot biomass. The regression between the shoot biomass (g·plant^−1^) and the N concentration (mg·g^−1^ DM) was conducted with the data obtained from the N-limiting growth treatments. The Nc value at the sampling point is calculated using the corresponding shoot biomass obtained from the non-N-limiting growth treatment. Furthermore, the NNI of the pakchoi at each sampling date was determined by dividing the total N concentration of the shoot (Nt) by Nc (NNI = Nt/Nc) based on a previous report on corn [53].

### 2.6. Model Development

#### 2.6.1. Feature Selection

The feature values were normalized using the SPSS 16.0 software. A one-way ANOVA was applied to screen the features acting as an optimal set of explanatory variables for model development. Features with *P* < 0.05 (Duncan method) at all the sampling dates were sent to a feature set to develop the model. 

#### 2.6.2. Models for Predicting Plant N Nutrition

Based on the screened phenotypic features, three models, namely the random forests (RF), support vector regression (SVR), and neural network (NN), were developed to quantitatively predict the NNI of the pakchoi. For the model development, we referred to the study by Guo et al. [43] with some modification. The three modeling algorithms were executed using the randomForest, nnet, and e1071 packages in R language (release 3.4.1), respectively. The RF model was executed using the randomForest package. The number of trees (ntree) and the number of features randomly sampled as candidates at each split (mtry) were used to find the best feature. In this study, the RF model was trained with ntree as 300 and mtry as 2, 5, and 8. For the SVR, we used the e1071 R package, which provides functionalities to use the libsvm library. The SVR model was trained with a kernel parameter C (from 0.00390625, 0.0078125, 0.015625, 0.03125, 0.0625, 0.125, 0.25, 0.5, 1, 2, 4, 8, 16, 32, 64, 128 and 256) and a regularization parameter gamma (from 0.00390625, 0.0078125, 0.015625, 0.03125, 0.0625, 0.125, 0.25, 0.5, 1, 2, 4, 8, 16, 32, 64, 128 and 256) in this study. The best combination of C and gamma leading to the highest prediction accuracy was chosen. For the NN, a nnet package was used to train the single-hidden-layer feed-forward neural network. Two main parameters, namely the decay and size, were tuned for the NN model in R. In this study, the decay was tuned from 0, 0.1, to 0.01, and the size was tuned from 2, 5, to 9. 

The total number of data samples in the dataset was 382. The dataset was randomly divided into a training set (75% of data) and a testing set (25% of data). The training data set was used for model development. The models were developed using the 10-fold cross-validation method. Among the training dataset, 10% of the data were used as validation in each cross-validation step. The root-mean-square error (RMSE) was used to select the optimal model.

The contribution weights of each feature to the regression model was also present. we chose “%IncMSE” (an increase in the mean squared error) to represent the criteria of relative importance in the RF model. The SVR and NN algorithms provide functionalities to use the corresponding library to measure the feature contribution of the models.

#### 2.6.3. Model Validation

To evaluate the performance of the regression models, we predicted the NNI for the testing set using the corresponding model. The actual NNI was measured destructively using Elementar. The prediction accuracy of the model was assessed in terms of the RMSE, adjusted determination coefficient (R^2^), and mean absolute error (MAE). 

### 2.7. Model Evaluation in Different Scenarios

To investigate the robustness and applicability of the three models under different conditions, the test dataset was reorganized into different scenarios, different growth stages (seedling stage and harvest period), and N nutrition status (N excessive or N deficiency). The model performances in these scenarios were evaluated. The simulation values were obtained using the predictive models, and the coincidence degree was analyzed using the linear regression method of SPSS 16.0. The relationship between the measured NNI and the simulated NNI was assessed based on the criteria: (1) adjusted determination coefficient (R^2^), i.e., the percentage of variance of NNI of the models; (2) relative standard error (RE), i.e., the ratio of the standard error of regression estimate to the mean of the actual value; (3) accuracy, i.e., the linear correlation coefficient between the simulated and actual values [54]. 

## 3. Results 

### 3.1. Yield and Quality

After 42 days of cultivation under different N levels, the aerial parts of the pakchoi exhibited obvious growth differences (Table 2). The fresh weight significantly increased with N application. However, the pakchoi supplied with higher N had lower fresh weight under tested N levels. The phytochemicals in the leaf were also affected significantly (p < 0.05) by N application. N fertilization significantly increased the chlorophyll content and decreased the flavonoid content; however, no differences were found among the tested N treatments. The availability of N had a prominent effect on the nutrition quality. At harvesting, the nitrate content increased with the N application, and the degree of elevation is positively correlated to the nitrogen level. Similarly, the soluble protein content increased with the N application; however, the content after T1 treatment was significantly higher than those after the other two treatments.

### 3.2. Accumulation of Biomass and N Concentration

The accumulation of biomass and N content in the shoots of the pakchoi was measured. Table 3 lists the results. The biomass increased continuously with the extension of the growth cycle; however, it was regulated by the nitrogen application rate. During the 7–28 days after treatment, the T3-treated pakchoi accumulated the highest biomass; until the 35*th* day, the T2-treated pakchoi had the highest biomass; on the 42*nd* day, the T1-treated pakchoi had the highest biomass. Simultaneously, the response of plant N concentration to N application was observed on the 7*th* day. However, the N concentration did not exhibit any significant difference between the N treatments until the 28*th* day, and it was always higher than that in the blank control. On the 28*th* day, the N concentration positively correlated to the nitrogen application rate. On the 35*th* and 42*nd* days, the N concentration had no significant difference between T2 and T3; however, it was higher than that in T1.

### 3.3. Nitrogen Nutrition Index

Under the tested N application rates, the shoot biomass in the T1-treated pakchoi was lower than that in the T3-treated pakchoi during the previous 28 days. Therefore, T1 belonged to the N-limiting growth treatment, and the relationship between plant N concentration and the shoot biomass was established with the data in T1. The Nc value at the sampling point was calculated using the corresponding shoot biomass value of the T1 or T2-treated pakchoi, and the Nc values were 56.08, 63.36, 69.16, 72.14, 76.44, and 79.46 g/kg. Table 4 lists the calculated NNI of the pakchoi at each sampling date. 

### 3.4. Feature Selection

**Figure 4:**
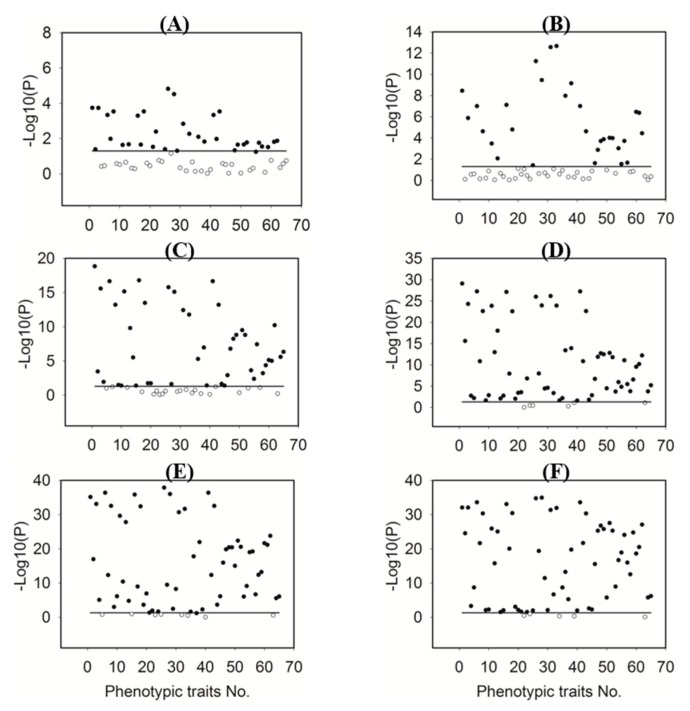
Significance analyses of phenotypic features with a p-value threshold of 0.05. The phenotypic features are indicated using numbers corresponding to their names. The solid horizontal line represents p = 0.05, i.e., −log10 (p) = 1.301. The solid and hollow circles indicate the conditions p < 0.05 and p > 0.05, respectively. (**A**), (**B**), (**C**), (**D**), (**E**), (**F**) corresponding to 7, 14, 21, 28, 35, and 42 days after the first N application, respectively.

In order to increase the efficiency of model development, phenotypic features selection is necessary to identify remarkable features which are relevant to plant N concentration. The significance of 65 extracted phenotypic features on classifying N application rates were investigated by using ANOVA (Figure 4). For better illustration, the p value was transformed into −log10 (p). There were 33, 29, 44, 59, 57, and 60 traits with p < 0.05 (i.e., −log10 (p) > 1.301) at corresponding sampling date, respectively. Finally, 23 phenotypic traits with P < 0.05 were selected at the six growth stages (G1–G6) to develop a model. The selected features contained 16 color-related (numbered as 1, 3, 6, 8, 11, 13, 16, 18, 26, 28, 31, 33, 36, 38, 41 and 43 in Table 1 respectively), three texture-related (numbered as 48, 48 and 51 in Table 1 respectively), and four geometry-related features (numbered as 52, 56, 61 and 62 in Table 1 respectively), and they are marked with asterisks in Table 1.

### 3.5. Model Development

The models were constructed to quantify the ability of image-based features in statistically predicting the NNI. We combined the NNI measurements with the phenotypic features and then divided them into a training dataset and a test dataset. A model was trained on the training dataset and then applied to the test dataset to predict the NNI. The models were developed using three widely used machine learning methods, and the RMSE was used to select the optimal model with the lowest values. The RF model was developed with ntree as 300 and mtry as 2. The SVR model was trained with a kernel parameter cost of 16 and a regularization parameter gamma of 0.03125. In the NN model, the decay and size were tuned to 0.01 and 5, respectively. Cross-validation results of three modeling algorithms applied to the training set, and the values of RMSE ± SD were 0.083 ± 0.008, 0.076 ± 0.004 and 0.085 ± 0.010 for RF, SVR and NN, respectively. The results show that the SVR model exhibits the best predictive ability for NNI. 

### 3.6. Model Validation

**Figure 5:**
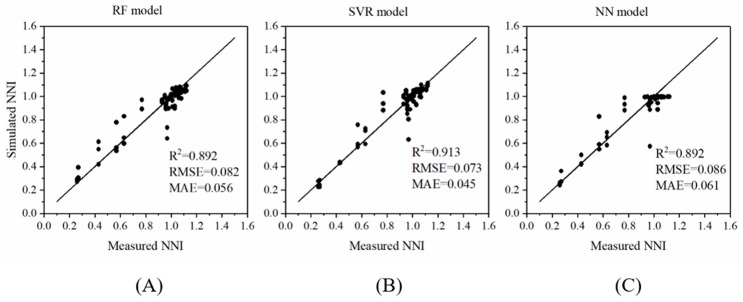
Quantitative relationship between simulated and measured nitrogen nutrition index (NNI) values, represented in the form of scatter plots of the manually measured NNI and the NNI predicted using three models: (**A**) random forest (RF), (**B**) support vector regression (SVR), and (**C**) neural network (NN). The line (y = x) represents the expected prediction. The quantitative relationship between the image-based features and the NNI was evaluated in terms of the coefficient of determination R^2^, root-mean-square error (RMSE), and mean absolute error (MAE) of the models.

The relationship between the simulated NNI (predicted using the regression models) and the measured NNI was assessed (Figure 5). The goodness of fit was quantified by R^2^, whereas the predictive ability was evaluated in terms of the RMSE and MAE. The R^2^ values of the three models reached approximately 0.900, showing an excellent goodness of fit. The RMSEs of the RF, SVR, and NN models were 0.082, 0.073, and 0.086, respectively, whereas the MAE values were 0.056, 0.045, and 0.061, respectively, indicating the effectiveness of predicting the plant NNI based on the phenotypic features. Among the three modeling algorithms, the SVR exhibited the lowest RMSE and MAE values, indicating that the SVR model predicted the NNI most accurately. 

### 3.7. Model Evaluation

#### 3.7.1. Model Evaluation under Different Plant N Nutrition Status

To check whether our models can be generalized across different scenarios, we applied the models under N surplus and deficiency conditions to predict the NNI. The R^2^, RE, and accuracy were selected as the evaluation indicators. Table 5 lists the evaluation results. Under N surplus condition, the R^2^ and accuracy of the three models are very low; however, the RE is very low. The performances of the models in predicting excessive N in the pakchoi were poor. Under N deficiency, the R^2^ of the three models crossed 0.90, and the accuracy reached approximately 0.95. However, the RE value of only the RF model was lower than 10%, indicating that the performance of this model was the best.

#### 3.7.2. Model Evaluation under Different Growth Stages of Pakchoi

We observed the model fitness under the seedling stage and harvest period simultaneously. The results (Table 5) showed the good predictive ability of the three models regardless of the growth stage. During the seedling periods, the R^2^ values of the three models ranged from 0.674 to 0.795, RE was lower than 10%, and accuracy was higher than 0.75. This shows that the prediction models can be used to guide the topdressing of N during the pakchoi growth cycle. At harvest, the R^2^ values of the three models reached above 0.95, RE was lower than 6.5%, and accuracy was higher than 0.85, once again exhibiting an excellent prediction performance. The precise prediction at harvest can help assess the N uptake by the current crops. Considering the above three evaluation factors, the RF model performed best in predicting the NNI regardless of the stage (seedling stage or harvest stage). 

### 3.8. Relative Importance of Phenotypic Traits in Predicting Plant NNI

**Figure 6:**
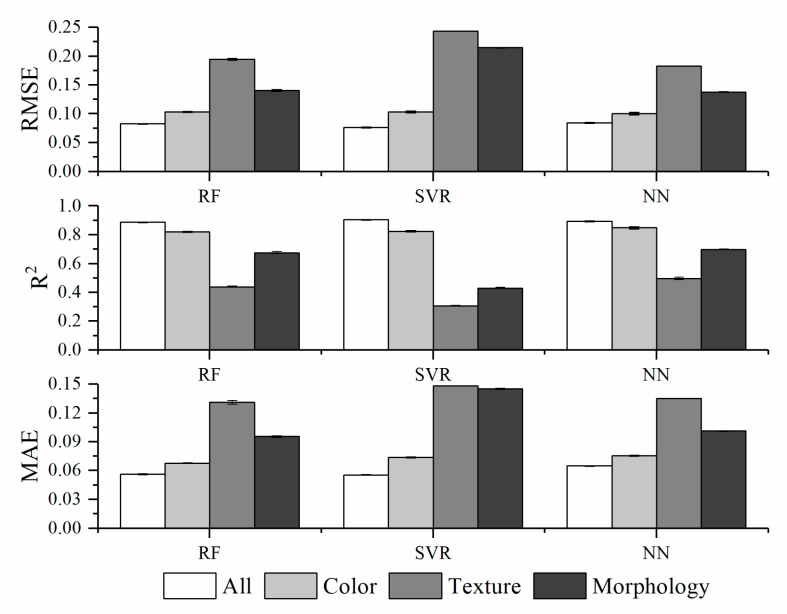
Capabilities of different image-based phenotyping methods in predicting the plant N status based on the evaluation of nitrogen nutrition index (NNI). The overall prediction accuracies of each type of phenotypic trait are included. The error bars represent the standard deviation. The white, light gray, gray, and dark gray bars indicate the prediction accuracies obtained using all traits, color-, texture-, and morphology-related traits, respectively.

As mentioned previously, the image-based features can be broadly classified into three categories: color-, texture-, and morphology-related traits. For each type of trait, we constructed a degenerate model using the corresponding traits as the predictors and compared the capability of each trait in predicting the NNI. The developments of three degenerate models were just based on the selected features (marked with asterisks in Table 1). Figure 6 shows the results. The color-related traits exhibited the best predictive performance among the three categories; however, they showed slightly lower performance than the full model in which all the traits were considered. Interestingly, the predictability of the other types of traits (such as the texture- and morphology-related traits) was substantial, indicating that these traits may act as unforeseen factors in NNI prediction. 

**Figure 7:**
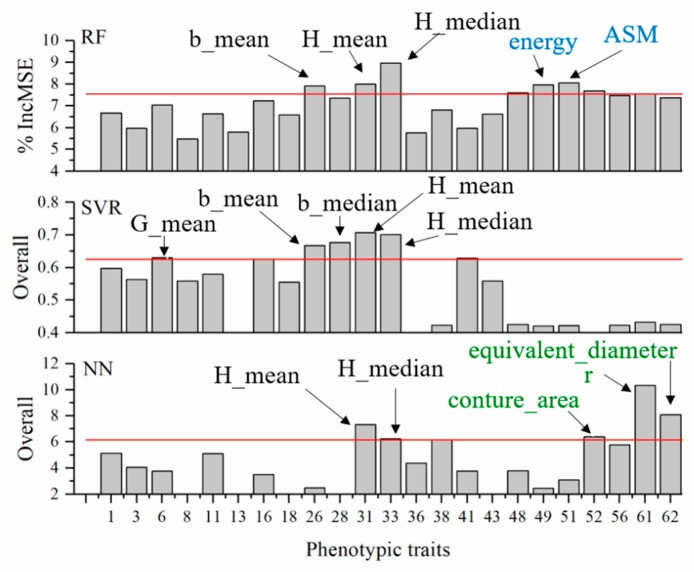
Relative contributions of individual features in predicting nitrogen nutrition index (NNI). Note that the phenotypic features are shared in the three panels. Phenotypic features are indicated in numbers corresponding to their names. The top five most important features are highlighted and labeled; the black, blue, and green fonts represent the color-, texture-, and morphology-related features, respectively.

We investigated the contribution of each feature in predicting the NNI using the full model (Figure 7). In the RF model, the contribution of a feature was determined as an increase in the prediction error (%IncMSE) when phenotypic data for this feature are permuted, thus indicating the contribution of the feature after considering its intercorrelation in the model. We found that the top five most important traits in the RF model in predicting NNI included both color and texture-related features. In the SVR and NN models, the relative importance of each trait was provided in the algorithms themselves. We found that the top five most important traits in predicting NNI only included color-related traits in the SVR model and color- and morphological-related traits in the NN model.

## 4. Discussion

### 4.1. Strategies for Integral Control of Water and Fertilization for Crops to Improve N Absorption and Utilization

Crop management practices such as the application of N fertilizer and alternative irrigation systems are often practiced to improve N absorption in crops. Management decisions, such as the appropriate selection of fertilization method and nitrogen application rate, are vital for improving the yield and quality in vegetable production systems. The results of this study showed that the yield and soluble protein content increased, whereas the nitrate content decreased under T1 treatment, indicating that the optimal fertilization strategy of low amount and high frequency could help reduce the nitrogen fertilizer input while ensuring the yield and quality of pakchoi. In a conventional fertilization method, a basal fertilizer was employed; however, the topdressing was ignored, leading to a significant amount of residual N in the soil [4]. The conventional strategies for N application increase the input and the loss of N, and they can cause nitrate pollution in groundwater and farmland [2,55,56], which is not favorable to a sustainable agricultural production.

The plant N concentration in field crops has been related to the aerial biomass as follows: $Nc=aDM^{-b}$, the parameter a represents the N concentration in the shoot biomass (t·ha^−1^), and the parameter b represents the coefficient of dilution describing the relationship between N concentration and shoot biomass [5,10]. Various studies have indicated that plant N concentration decreases with the growth cycle. However, the plant N concentration in pakchoi remained high and even increased with the accumulation of shoot biomass, probably because of the fertilization strategy of low amount and high frequency in the pot experiment. This fertilization strategy might promote N absorption and utilization, leading to a faster accumulation of N than shoot biomass. Therefore, the relationship between Nc and the aerial biomass is related to the N strategy. Different critical nitrogen concentration curves should be established for different nitrogen fertilization strategies applied to the crop. These results reveal that topdressing could promote the absorption of N fertilizer by plants and contribute to the reduction in N fertilization.

### 4.2. Accuracy Comparison of Full Model with the Color Model in Predicting Plant Nitrogen

Three types of phenotypic features (color, texture, and geometry-related features) based on visible light imaging were extracted, and the capability of each feature in predicting the NNI was compared. The color-related traits showed the best predictive performance among the three categories for NNI prediction, which might be attributed that the number of color features is 4 times bigger than the number of textures and geometry (16 features vs. 3 features). However, our result was in agreement with Rorie et al. [57] who found a close relationship between a dark green color index and leaf N concentration. The association between the color features and leaf N is mainly due to phytochemicals such as chlorophyll. Thus, leaf N prediction can be performed by non-destructive chlorophyll measurement. However, chlorophyll meters only provide a single point measurement; they cannot work at high resolutions and cannot provide proper information about the spatial structures of the product [48]. In addition, our results showed that the texture-related and morphology-related traits exhibited a good NNI predictability, and a significant difference was observed (P < 0.05) in phenotypic features including color, texture, and morphology. This indicates that there exist limitations in the conventional evaluation of plant nitrogen nutrition status based on only the leaf color features, and that the ability of the texture- and geometry-related traits to predict plant N cannot be ignored.

In this study, the color, texture, and morphological-related features were used as input parameters. RF, SVR, and NN models were developed to evaluate the NNI of pakchoi. The R^2^ values of the three models reached approximately 0.900, and the RMSE value was lower than 0.1. The results revealed that phenotypic imaging combined with a machine learning algorithm can be used to effectively evaluate the nitrogen nutrition status of plants.

### 4.3. Random Forest (RF) Model based on Phenotyping Is the Most Robust in Predicting NNI 

Many algorithms exist in the field of machine learning, each having different principles. Thus, the contribution of the same phenotypic feature in the different algorithms can be different. In the top five most contributing features, the RF model included H_median, ASM, H_mean, energy, and b_mean; the SVR model included H_mean, H_median, b_median, b_mean, and G_mean; the NN model included r, equivalent_diameter, H_mean, conture_area, and H_media. Different algorithms have different dependences on the same phenotypic features, resulting in the local adaptability of each algorithm. Therefore, to select an appropriate NNI model under different scenarios, such as growing periods and soil fertility, it is necessary to determine the robustness and applicability of each algorithm.

In our research, the evaluation effects of the three models were compared under different conditions. The results showed that none of the models could effectively predict the nutritional status when the plant N is excessive, indicating that camera vision in the visible range has a poor resolution when the plant N concentration is close to the normal range. Our results are in agreement with those of Tei et al. [58] who concluded that plants with surplus N reflect less visible light than N-deficient plants. Plateau responses occurred at relatively high N contents, suggesting saturation. However, it is unclear under what exact conditions saturation would occur [59,60]; this requires further elucidation. It is necessary to choose other optical sensors or non-destructive testing techniques to evaluate the N surplus status of a plant. For example, Padilla et al. [22] found that chlorophyll readings and canopy reflectance indices could sensitively respond to the absorption of N by muskmelon. Under N-deficit conditions, the RF model performed best with higher R^2^ and lower RE values. We assessed the NNI prediction models throughout the entire growth cycle of pakchoi. The precise prediction of NNI at the seedling stage indicated that the prediction models can be used to guide N topdressing. The precise prediction of NNI at the harvest stage is beneficial for assessing the N uptake and calculating the N consumption by the current crops. The RF model exhibited outstanding robustness and applicability under different scenarios and thus can be used in practice.

## 5. Conclusions

Twenty-three phenotypic features related to plant N were screened, and a quantitative relationship between the NNI and the image-based phenotypic features was established using the following algorithms: random forest (RF), support vector regression (SVR), and neural network (NN). The three models exhibited excellent fitting and predictive ability. The RF full model was more robust, with NNI prediction accuracies reaching 0.823 and 0.943 at the seedling and harvest stages, respectively. The highly accurate prediction of NNI based on this model allows a rapid and low-cost method to diagnose plant N nutrition, relieving the phenotyping bottleneck in plant N measurement in the field of precision crop management.

## Figures and Tables

**Table 1:** Phenotypic features extracted from images in pakchoi.

Category	Trait Name	Description	No.
Color	*R_mean, R_std, *R_median, R_ range, R_coefficient of variation	Statistics in the red range of the RGB color space	1–5
	*G_mean, G_std, *G_median, G_ range, G_coefficient of variation	Statistics in the green range of the RGB color space	6–10
	*B_mean, B_std, *B_median, B_ range, B_coefficient of variation	Statistics in the blue range of the RGB color space	11–15
	*L_mean, L_std, *L_median, L_ range, L_coefficient of variation	Statistics of L channel in LAB color space	16–20
	a_mean, a_std, a_median, a_ range, a_coefficient of variation	Statistics of A channel in LAB color space	21–25
	*b_mean, b_std, *b_median, b_ range, b_coefficient of variation	Statistics of B channel in LAB color space	26–30
	*H_mean, H_std, *H_median, H_ range, H_coefficient of variation	Statistics of H in HSV color space	31–35
	*S_mean, S_std, *S_median, S_ range, S_coefficient of variation	Statistics of S in HSV color space	36–40
	*V_mean, V_std, *V_median, V_ range, V_coefficient of variation	Statistics of V in HSV color space	41–45
Texture	contrast	Definition and grooving depth of texture	46
	dissimilarity	The difference of grey scale	47
	*homogeneity	The local changes of image texture	48
	*energy	The degree of thickness and uniformity for texture	49
	correlation	The correlation of the local grey scale	50
	*ASM	Angular second moment	51
Morphology	*contour_area	Area of plant contour	52
	perimeter	The length of plant contour	53
	w	The width of the bounding box	54
	h	The height of the bounding box	55
	*hull_area	Convex hull area (mm^2^)	56
	_w	The width of the minimum circumscribed rectangle	57
	_h	The height of the minimum circumscribed rectangle	58
	MA	The macro axis of the ellipse	59
	ma	The minor axis of the ellipse	60
	*r	The radius of the minimum circumscribed circle	61
	*equivalent_diameter	The diameter of a circle equal to the contour area	62
	aspect_ration	The width-height ratio of the bounding box	63
	extent	The area ratio between contour and bounding box	64
	solidity	The area ratio between contour and convex hull	65

Note:(1) _mean, _std, _median, _range, and _coefficient of variation represent mean, standard deviation, median, range and coefficient of variation, respectively. (2) The traits marked with asterisks were selected to develop a model. (3) RGB (Red, Green, Blue), LAB ( L is luminosity, A is the range from magenta to green, and B is the range from yellow to blue, and HSV ( Hue, Saturation, Value)

**Table 2:** Yield and quality of pakchoi after 42 days of cultivation.

Treatments	Yield (g·plant^−1^)	Chlorophyll (μg·cm^−2^)	Flavonol Index	Nitrate (mg·kg^−1^ FW)	Soluble Protein (mg·g^−1^ FW)
CK	6.04 ± 0.45d	28.70 ± 1.67b	1.37 ± 0.26a	200.92 ± 3.51c	5.21 ± 0.58c
T1	27.65 ± 0.28a	32.98 ± 4.98a	0.74 ± 0.11b	271.47 ± 23.35b	35.10 ± 1.97a
T2	23.88 ± 0.04b	32.17 ± 2.78a	0.80 ± 0.11b	298.31 ± 19.16b	31.63 ± 1.91b
T3	21.37 ± 0.34c	33.08 ± 2.75a	0.78 ± 0.11b	364.25 ± 25.34a	30.47 ± 2.61b

Note: Data of the table represent average value ± standard deviation (n = 3) and those with the different letters in the same column are significantly different (*p* < 0.05).

**Table 3:** Accumulation of dry weight (g·plant^−1^) and nitrogen concentration (g·kg^−1^ DW) in shoot.

Index	Treatment	Days after Transplant (d)					
		7	14	21	28	35	42
Biomass	CK	0.065 ± 0.007b	0.161 ± 0.006b	0.186 ± 0.008c	0.338 ± 0.012c	0.479 ± 0.037b	0.617 ± 0.037c
	T1	0.067 ± 0.004b	0.174 ± 0.019b	0.293 ± 0.010b	0.580 ± 0.023b	0.973 ± 0.019a	1.363 ± 0.032a
	T2	0.080 ± 0.004a	0.189 ± 0.023b	0.306 ± 0.014b	0.574 ± 0.018b	0.994 ± 0.051a	1.280 ± 0.025b
	T3	0.079 ± 0.010a	0.215 ± 0.011a	0.440 ± 0.025a	0.620 ± 0.014a	0.970 ± 0.017a	1.282 ± 0.027b
Nitrogen concentration	CK	43.30 ± 2.31b	39.70 ± 2.76b	39.07 ± 1.59b	30.80 ± 1.57d	20.50 ± 1.08c	20.50 ± 0.79c
	T1	57.50 ± 0.26a	61.27 ± 1.01a	69.63 ± 1.90a	67.30 ± 0.17c	72.70 ± 2.66b	83.77 ± 2.57b
	T2	57.63 ± 0.85a	62.20 ± 1.23a	74.87 ± 2.25a	71.97 ± 0.99b	81.87 ± 1.65a	88.07 ± 2.67ab
	T3	60.57 ± 1.27a	64.00 ± 0.85a	73.30 ± 4.98a	76.63 ± 0.50a	79.47 ± 0.74a	88.80 ± 2.11a

Note: Data of the table represent average value ± standard deviation (n = 3) and those with the different letters in the same column are significantly different (p < 0.05).

**Table 4:** Nitrogen nutrition index (NNI) in pakchoi at different nitrogen treatments.

Treatment	Days after Transplant (d)					
	7	14	21	28	35	42
CK	0.77	0.63	0.57	0.43	0.27	0.26
T1	1.03	0.97	1.01	0.93	0.95	1.05
T2	1.03	0.98	1.08	1.00	1.07	1.11
T3	1.08	1.01	1.06	1.06	1.04	1.12

Note: NNI > 1, excessive nitrogen nutrition; NNI = 1, optimal nitrogen nutrition; NNI < 1, deficient N nutrition.

**Table 5:** Model evaluation result under different plant N nutrition status and growth stages.

Different Scenarios	Range of Measured NNI	Model	Model Evaluation Results			
			Range of Simulated NNI	R^2^	Relative Error (%)	Accuracy
Excessive	1.01~1.12	**RF**	**0.895~1.101**	**0.470**	**2.57**	**0.787**
		SVR	0.772~1.135	0.206	3.56	0.586
		NN	0.822~1.000	0.016	1.76	0.085
Low	0.26~0.93	**RF**	**0.262~1.081**	**0.945**	**8.35**	**0.948**
		SVR	0.173~1.087	0.921	10.51	0.984
		NN	0.237~1.000	0.918	10.35	0.952
Seedling period	0.63~1.08	**RF**	**0.495~1.068**	**0.795**	**6.56**	**0.823**
		SVR	0.348~1.084	0.703	8.79	0.856
		NN	0.416~1.000	0.674	8.35	0.766
Harvest period	0.26~1.12	**RF**	**0.262~1.101**	**0.985**	**4.49**	**0.943**
		SVR	0.173~1.135	0.981	5.23	0.974
		NN	0.237~1.000	0.969	6.06	0.869

Note: RF, Random Forest; SVR, Support Vector Regression; NN, Neural Network

## Supplementary Materials

The following are available online at https://www.mdpi.com/1424-8220/19/11/2448/s1.
