# Supplementary material for: Non-Invasive Sensing of Nitrogen in Plant Using Digital Images and Machine Learning for Brassica Campestris ssp. Chinensis L
Source: Sensors (Basel). 2019 May 29;19(11):2448. doi: 10.3390/s19112448 (PMC6603544; doi:10.3390/s19112448)
Supplement: Supplementary file 1 [file sensors-19-02448-s001.pdf]

sensors

MDPI

Supplementary Material

The details of image processing

The image analysis was implemented by using the OpenCV (3.4.4 version) and the Python language (3.6.1). We use the following steps for robust image processing.

(1) Excessive green index (EGI) calculation；the original images were transformed by using 2G-R-B, and the two thresholds (where 40 identified the minimum value and 200 the maximum value) were set to identify the green part of the images (including plant and the green area of background). The processing flow as shown below.

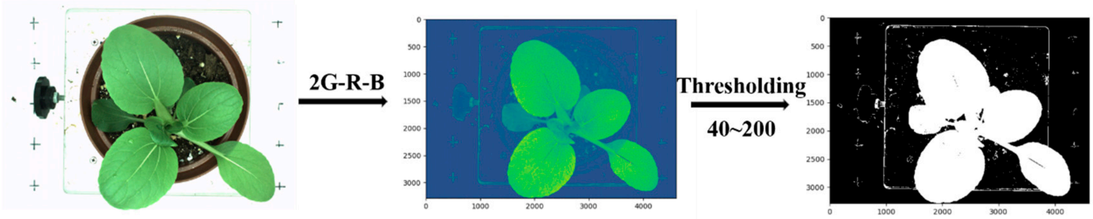

(2) Grayscale processing; The original images were pretreated by graying, and then the threshold value segmentation was used for removing the high brightness area. The grayscale value was set as 240. The processing flow as shown below.

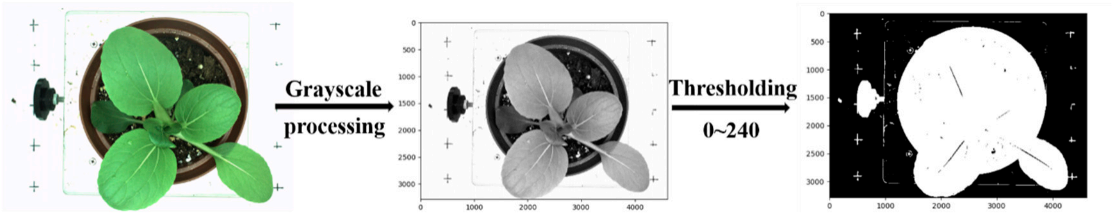

(3) Intersection calculation; we take the intersection of the mask obtained by the two operations above, and then the noise (small size) was eliminated based on the mathematical morphological open operation. The processing flow as shown below.

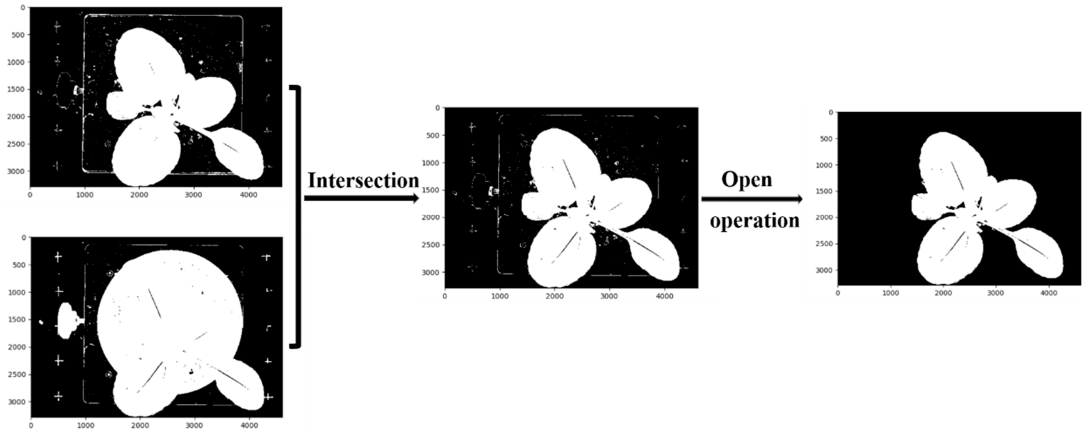

(4) Denoising; The operations of “remove the small objects” and “remove small hole” were done by using OpenCV to remove the residual noise and restore leaf veins deleted, respectively. Hence, the final images were acquired, and it was shown below.

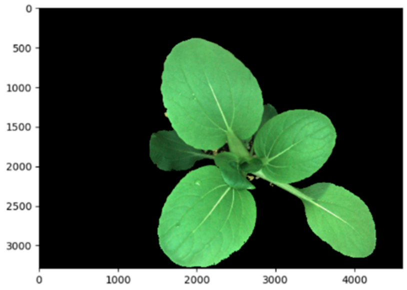

The formulas of texture traits

The texture is a law describing the spatial distribution of gray scale in the neighborhood of blade image. The texture traits were extracted over the whole image, and the extraction process includes two steps as below.

First, the final images were processed by graying, and grey-level co-occurrence matrix (GLCM) was computed. The GLCM is a 2D matrix that captures how often the neighbor values A and B occur in an image. Look at the GLCM as a matrix of N x N dimensions that captures the amplitude response of the reference position in the columns and the amplitudes of the neighboring position in the rows. N is the range of all values the data can have.

Secondly, the texture attributes were computed according to the following formulas, where levels are equal to N values, where i represents some value on N rows, j represents some value on N columns, $P_{i,j}$ represents the probability that the row is i and the column is j.

$$\text{Contrast} = \sum_{i,j=0}^{N} P_{i,j}(i-j)^2$$

$$\text{Dissimilarity} = \sum_{i,j=0}^{N-1} P_{i,j}\,|i-j|$$

$$\text{Homogeneity} = \sum_{i,j=0}^{N-1} \frac{P_{i,j}}{1 + (i - j)^2}$$

$$\text{ASM} = \sum_{i,j=0}^{N-1} P_{i,j}^2$$

$$\text{Energy} = \sqrt{ASM}$$

$$\mu_i = \sum_{i,j=0}^{N-1} i(P_{i,j}) = \mu_j = \sum_{i,j=0}^{N-1} j(P_{i,j})$$

$$\delta_i^2 = \sum_{i,j=0}^{N-1} P_{i,j}(i - \mu_i)^2 = \delta_j^2 = \sum_{i,j=0}^{N-1} P_{i,j}(j - \mu_j)^2$$

$$\text{Correlation} = \sum_{i,j=0}^{N-1} [\frac{(i - \mu_i) \quad (j - \mu_i)}{\sqrt{(\sigma_i^2) \quad (\sigma_j^2)}}]$$

© 2019 by the authors. Submitted for possible open access publication under the terms and conditions of the Creative Commons Attribution (CC BY) license (http://creativecommons.org/licenses/by/4.0/).
